# Does radiotherapy still have a role in unresected biliary tract cancer?

**DOI:** 10.1002/cam4.975

**Published:** 2016-11-28

**Authors:** Erqi L. Pollom, Muthuraman Alagappan, Lesley S. Park, Alice S. Whittemore, Albert C. Koong, Daniel T. Chang

**Affiliations:** ^1^ Department of Radiation OncologyStanford University School of MedicineStanfordCalifornia; ^2^ Department of Health Research and PolicyStanford University School of MedicineStanfordCalifornia

**Keywords:** Chemoradiation, cholangiocarcinoma, elderly, gallbladder, SEER‐Medicare

## Abstract

The benefits of radiotherapy for inoperable biliary tract cancer remain unclear due to the lack of randomized data. We evaluated the impact of radiotherapy on survival in elderly patients using the SEER‐Medicare database. Patients in the SEER‐Medicare database with inoperable biliary tract tumors diagnosed between 1998 and 2011 were included. We used multivariate logistic regression to evaluate factors associated with treatment selection, and multivariate Cox regression and propensity score matching to evaluate treatment selection in relation to subsequent survival. Of the 2343 patients included, 451 (19%) received radiotherapy within 4 months of diagnosis. The use of radiotherapy declined over time, and was influenced by receipt of chemotherapy and patient age, race, marital status, poverty status, and tumor stage and type. Median survival was 9.3 (95% CI 8.7–9.7) months among patients who did not receive radiation and 10.0 (95% CI 9.1–11.3) months among those who received radiation, conditional on having survived 4 months. In patients who received chemotherapy (*n* = 1053), receipt of radiation was associated with improved survival, with an adjusted hazard ratio of 0.82 (95% 0.70–0.97, *P* = 0.02). In patients who did not receive chemotherapy (*n* = 1290), receipt of radiation was not associated with improved survival, with an adjusted hazard ratio of 1.09 (95% 0.91–1.30, *P* = 0.34). Propensity‐scored matched analyses showed similar results. Despite the survival benefit associated with the addition of radiotherapy to chemotherapy, the use of radiation for unresectable biliary tract cancers has declined over time.

## Introduction

Biliary tract tumors arise in either the intrahepatic or extrahepatic bile ducts or the gallbladder and are associated with an aggressive disease course. The majority of patients presents with advanced disease at the time of diagnosis and are not eligible for surgical resection 1, 2, 3. The prognosis of inoperable patients is poor, with a median survival of only 3.9 months for those undergoing supportive care alone 4. Although chemotherapy is an accepted standard of care for inoperable patients 5, 6, 7, 8, the role of radiation is less well‐defined 9.

Radiotherapy is often used to prevent or delay local‐regional progression, which can be associated with significant hepatobiliary‐related morbidity and even death. Radiotherapy has been shown to reduce pain and improve bile duct patency in patients with inoperable biliary tract cancers 10. However, data regarding the survival benefit with radiation is conflicting and limited to mostly nonrandomized series due to the rarity of this disease 10, 11, 12, 13, 14, 15, 16, 17, 18, 19, 20, 21, 22. Additionally, biliary tract cancer primarily affects the elderly 23, 24, which makes estimating treatment effect even more challenging as trials often exclude elderly patients.

The availability of large datasets has provided opportunities to compare the effectiveness of different treatment modalities in complete populations of cancer patients. Prior studies using the Surveillance, Epidemiology, and End Results (SEER) database found a large survival benefit with the use of radiotherapy in patients with intrahepatic and extrahepatic cholangiocarcinomas, but did not account for comorbidity, the use of chemotherapy, or immortal time bias, which occurs in observational studies when patients have to be alive in order to receive a treatment, thus biasing survival in favor of treatment 14, 15. In this study, we use the SEER‐Medicare database to evaluate the patterns of utilization of radiotherapy in elderly patients with inoperable biliary cancer, and to assess the impact of the utilization of radiotherapy on survival.

## Methods

### Study overview

We performed a retrospective cohort study using the 2014 linkage of the SEER‐Medicare database to examine the utilization and impact of radiation on survival outcomes in elderly patients with unresected biliary tract tumors. Additional details about this database can be found in Appendix A. The Institutional Review Board of Stanford University deemed this study exempt from review.

### Cohort selection

We identified patients with invasive biliary tract tumors diagnosed between 1998 and 2011 who did not undergo resection. We restricted our cohort to patients who had continuous enrollment in Medicare Part A and Part B and no health‐maintenance organization coverage during the 16‐month period from 12 months prior to diagnosis to 4 months after diagnosis. This restriction allowed ascertainment of comorbidity status as well as initial treatment. In addition, this restriction excluded patients who died within 4 months of diagnosis, addressing potential immortality bias 25, 26, 27, 28, 29. Patients who died within this time would not have had the opportunity to undergo and benefit from radiation. This interval was chosen a priori to be broad enough after diagnosis to allow receipt of radiotherapy, but narrow enough so as to avoid counting radiation courses for disease progression or recurrence. Details of our cohort selection are available in Appendix A.

### Primary exposure variable

We determined radiotherapy administration from Medicare claims (Appendix B). We considered a patient to have received radiotherapy at diagnosis only if the first radiation delivery claim pertained to within 1 month prior to and 4 months after diagnosis date.

### Study covariates

We used the SEER database to obtain demographic, disease, and socioeconomic characteristics. Because of variations in staging across tumor types and over the time period of our study as well as lack of definitive, surgical staging, we used SEER historic staging for consistency. We used state‐buy‐in status as an indicator of poverty. Medicare buy‐in benefits, operated by Medicaid, help low‐income Medicare beneficiaries pay their Medicare premiums, deductibles, and copayments. We created a composite measure for area socioeconomic status based on variables from the 2000 US census data: median household income, percentage of persons 25 years of age or older with at least a high school education, and percentage of people below the poverty level 30. We constructed the composite measure by classifying into quartiles the sum of the *z*‐scores for the variables. We used the Area Resource File to determine radiation oncologist density in the health services area (HSA) to which each patient belonged. The density of radiation oncologists per HSA was determined by dividing the number of radiation oncologists by the Medicare eligible population for a given HSA and categorized into quartiles.

We used linked claims data to obtain comorbidity/performance status and interactions with the healthcare system. We calculated a modified Charlson comorbidity index using inpatient and outpatient claims for an interval before cancer diagnosis of 12 months to 1 month 31, 32, 33, 34, 35. We used a validated measure of predicted poor disability status as a claims‐based proxy for poor performance status 36, 37.

We identified biliary stent/drain placement and chemotherapy administration from Medicare claims using previously described methods (Appendix B) 38, 39. As with radiation, we only considered chemotherapy and biliary/drain placement claims within 1 month prior to and 4 months after diagnosis date.

### Primary outcome

Our primary outcome was all‐cause mortality and was determined based on SEER dates of death according to Social Security Administration data, with follow‐up through December 31, 2013. Survival time was defined as the time from cancer diagnosis date to date of death or December 31, 2013, whichever came first. Persons surviving past this date were censored. For cancer‐specific mortality 40, we used the SEER variable designating whether a person had died of their cancer, based on cause of death on death certificates. Persons who died from noncancer causes were censored.

### Statistical analysis

Bivariate associations between covariates and receipt of radiotherapy were evaluated using Pearson chi‐square tests. We also used multivariate logistic regression models to evaluate associations between receipt of radiotherapy and patient characteristics. We used a reverse, stepwise selection process to construct a working model, retaining variables with *P* < 0.1.

We assessed the relation between radiation treatment and all‐cause and cancer‐specific mortality using univariate and multivariate Cox proportional hazard models. Predictors were checked for departures from the proportional hazards assumption visually and using Schoenfeld residuals 41.

Because radiation treatment was not randomly assigned in this patient population, we performed a confirmatory, propensity score analysis. We developed a logistic regression model including relevant covariates to calculate and assign propensity scores for radiation treatment to each patient. Patients were then matched on propensity score in order to balance the observed covariates. Additional details of the propensity‐score analysis are provided in Appendix C.

All statistical analyses were performed using SAS, version 9.4 (SAS Institute, Cary, NC).

## Results

### Study cohort characteristics and factors influencing treatment selection

Appendix A shows our cohort selection process. A total of 2343 patients met our inclusion criteria. Among these patients, 451 (19%) received radiotherapy within 4 months of diagnosis. Table 1 summarizes the baseline characteristics of our cohort.

**Table 1:** Baseline characteristics associated with receipt of radiation among patients with inoperable biliary tract cancers in SEER‐Medicare

Characteristic	Did not receive radiation	Received radiation	*P* a
Total cohort	1892 (81%)	451 (19%)	
Age at diagnosis			
≤70	496 (79%)	132 (21%)	0.0001
71–75	405 (76%)	126 (24%)	
76–80	401 (80%)	98 (20%)	
>80	590 (86%)	95 (14%)	
Gender			
Male	804 (78%)	229 (22%)	0.002
Female	1088 (83%)	222 (17%)	
Race			
White	1530 (81%)	357 (19%)	0.009
Black	167 (85%)	29 (15%)	
Other^b^^,^ c	195 (75%)	65 (25%)	
Marital status			
Married	913 (77%)	276 (23%)	<0.0001
Single^d^	918 (85%)	164 (15%)	
Unknown	61 (85%)	11 (15%)	
Comorbidity index			
0	997 (80%)	242 (20%)	0.86
1	483 (81%)	116 (19%)	
2+	412 (82%)	93 (18%)	
Disability status			
Good	1674 (80%)	423 (20%)	0.0009
Poor	218 (89%)	28 (11%)	
Tumor type			
Extrahepaticcholangiocarcinoma	837 (77%)	254 (23%)	<0.0001
Gallbladder	394 (89%)	50 (11%)	
Intrahepaticcholangiocarcinoma	661 (82%)	147 (18%)	
SEER historic stage			
Local	573 (82%)	130 (18%)	<0.0001
Regional	575 (73%)	210 (27%)	
Distant	744 (87%)	111 (13%)	
Year of diagnosis			
1998–2002	450 (77%)	136 (23%)	0.02
2003–2007	721 (82%)	159 (18%)	
2008–2011	721 (82%)	156 (18%)	
SEER registry^e^			
Midwest	252 (78%)	71 (22%)	0.37
West	856 (81%)	196 (19%)	
Northeast	416 (82%)	89 (18%)	
South	368 (79%)	95 (21%)	
SES composite index^e^			
1st quartile (lowest)^c^	493 (80%)	122 (20%)	0.49
2nd quartile	438 (79%)	115 (21%)	
3rd quartile	446 (81%)	105 (19%)	
4th quartile (highest)	515 (83%)	109 (17%)	
Rural/urban			
Metropolitan	1611 (81%)	389 (19%)	0.55
Urban/rural	281 (82%)	62 (18%)	
HSA radiation oncologist density			
1st quartile (lowest)	508 (82%)	112 (18%)	0.002
2nd quartile	430 (82%)	94 (18%)	
3rd quartile	419 (82%)	91 (18%)	
4th quartile(highest)	501 (79%)	132 (21%)	
Unknown	34 (61%)	22 (39%)	
State buy‐in			
Yes	495 (87%)	75 (13%)	<0.0001
No	1397 (79%)	376 (21%)	
Any chemotherapy			
Yes	779 (74%)	274 (26%)	<0.0001
No	1113 (86%)	177 (14%)	
Gemcitabine + Cisplatin chemotherapy			
Yes	158 (92%)	14 (8%)	0.0001
No	1734 (80%)	437 (20%)	
Stent/Drain			
Yes	1165 (77%)	352 (23%)	<0.0001
No	727 (88%)	99 (12%)	

No, number; SEER, Surveillance, Epidemiology, and End Results; SES, socioeconomic status; HSA, Health Services Area.

a Based on Pearson's chi‐square test.

b Other includes American Indian/Alaskan Native, Asian/Pacific Islander.

c Unknown included with this category for privacy purposes due to low number of patients.

d Single includes unmarried, divorced, separated, and widowed.

West: San Francisco, Hawaii, New Mexico, Seattle, Utah, San Jose, Los Angeles, Greater California; Midwest: Detroit, Iowa; Northeast: Connecticut, New Jersey; South: Atlantic, Rural Georgia, Kentucky, Louisiana, Greater Georgia.

e Composite measure for area socioeconomic status based on following variables from the 2000 US census data: median household income, percentage of persons 25 years of age or older with at least a high school education, and percentage of people below the poverty level.

Receipt of radiation treatment was associated with patient, tumor, and socioeconomic factors. In a multivariate model, age >80, single relationship status, metastatic stage disease, and having state buy‐in were significantly associated with omission of radiotherapy while American Indian/Alaskan Native/Asian/Pacific Islander race, extrahepatic cholangiocarcinoma, regional stage disease, presence of a biliary stent or drain, and receipt of chemotherapy were independent predictors of radiotherapy use within 4 months of diagnosis (Table 2).

**Table 2:** Factors independently associated with receipt of radiotherapy in multivariate logistic model

Characteristic	Adjusted OR for receipt of radiation	95% CI	*P*
Age at diagnosis			
≤70	Reference		–
71–75	1.14	0.84–1.54	0.40
76–80	0.94	0.68–1.29	0.68
>80	0.69	0.50–0.97	0.03
Race			
White	Reference		–
Black	0.90	0.57–1.42	0.65
Other^a^^,^ b	1.86	1.30–2.65	0.0006
Marital status			
Married	Reference		–
Single^c^	0.74	0.59–0.93	0.01
Tumor type			
Extrahepatic cholangiocarcinoma	Reference		–
Gallbladder	0.56	0.38–0.81	0.002
Intrahepatic cholangiocarcinoma	0.79	0.61–1.03	0.08
SEER historic stage			
Local	Reference		–
Regional	1.33	1.01–1.74	0.04
Distant	0.53	0.39–0.72	<0.0001
Year of diagnosis			
1998–2002	Reference		–
2003–2007	0.71	0.54–0.94	0.02
2008–2011	0.71	0.53–0.94	0.02
State buy‐in			
Yes	Reference		–
No	0.57	0.42–0.77	0.0003
Chemotherapy			
Yes	Reference		–
No	0.37	0.29–0.48	<0.0001
Stent/Drain			
Yes	Reference		–
No	1.98	1.49–2.63	<0.0001

OR, odds ratio; CI, confidence interval; SEER, Surveillance, Epidemiology, and End Results.

a Other includes American Indian/Alaskan Native, Asian/Pacific Islander.

b Unknown included with this category for privacy purposes due to low number of patients.

c Single includes unmarried, divorced, separated, and widowed.

With respect to patterns of therapy, the use of radiation declined over time, with its use declining from 28.6% in 1998 to 15.7% in 2011 (annual percent change of −0.8% per year, *P* = 0.002). Of the patients who received radiation, 20.6% received either intensity‐modulated (IMRT) or stereotactic body radiotherapy (SBRT). The majority of patients (67%) had their first radiation claim within 3 weeks of their first chemotherapy claim. The use of chemotherapy increased over time, with its use increasing from 36.9% in 1998 to 57.8% in 2011 (annual percent change of +1.5% per year, *P* < 0.0001).

### Impact of treatment on survival outcomes

Median survival was 9.3 (95% CI 8.7–9.7) months among patients who did not receive radiation and 10.0 (95% CI 9.1–11.3) months among those who received radiation (log‐rank *P* = 0.02).

**Figure 1:**
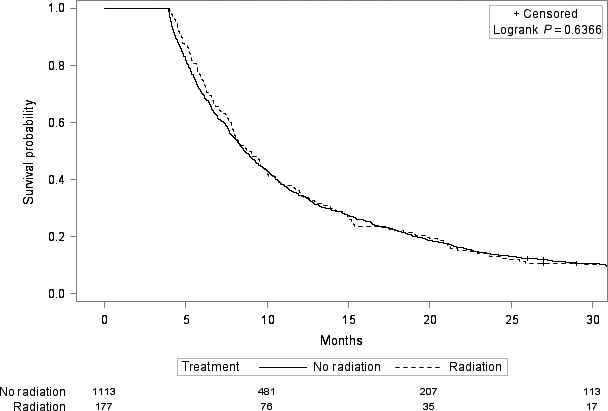
Kaplan–Meier survival curves of those who received radiation versus those who did not receive radiation in the no chemotherapy cohort.

**Figure 2:**
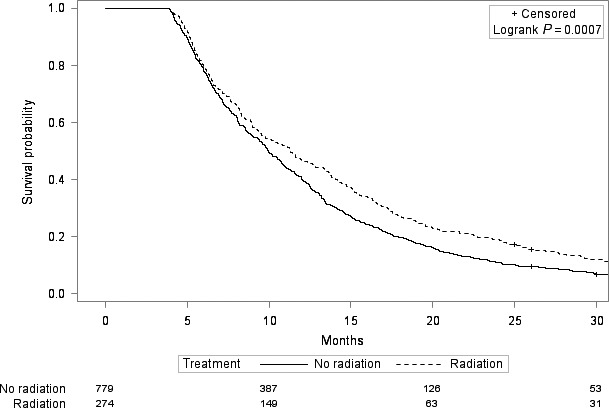
Kaplan–Meier survival curves of those who received radiation versus those who did not receive radiation in the chemotherapy cohort.

In univariate Cox regression, the receipt of radiation treatment was associated with improved overall survival, with a hazard ratio of 0.90 (95% CI 0.81–0.99, *P* = 0.04). After adjustment for demographic, tumor, patient, treatment, and socioeconomic characteristics (Table 1), radiation was no longer significantly associated with survival, with a hazard ratio of 0.95 (95% CI: 0.84–1.07, *P* = 0.38). However, when we tested additional interactions in the adjusted model, we found a significant interaction between radiation and chemotherapy (*P* = 0.02). In patients who received chemotherapy (*n* = 1053), receipt of radiation was associated with improved survival, with an adjusted hazard ratio of 0.82 (95% 0.70–0.97, *P* = 0.02). In patients who did not receive chemotherapy (*n* = 1290), receipt of radiation was not associated with improved survival, with an adjusted hazard ratio of 1.09 (95% 0.91–1.30, *P* = 0.34). Figures 1 and 2 show the Kaplan–Meier curves in the chemotherapy and no chemotherapy cohorts.

Other factors that were significantly associated with worse overall survival in the multivariate Cox regression were older age and more advanced tumor stage (Table 3). There was no evidence that the radiation‐survival association differed by tumor type (intrahepatic cholangiocarcinoma, extrahepatic cholangiocarcinoma, or gallbladder cancer) in either the patients who received chemotherapy (*P* = 0.50 for interaction) or the patients who did not receive chemotherapy (*P* = 0.20 for interaction). However, within the chemotherapy cohort, there was an interaction between radiation and tumor stage (*P* = 0.04). Patients who had metastatic disease and received chemotherapy did not appear to derive a survival benefit from radiation with an adjusted hazard ratio of 1.12 (95% CI 0.85–1.46, *P* = 0.43). Patients who did not have metastatic disease and received chemotherapy had improved survival with radiation, with an adjusted hazard ratio of 0.75 (95% CI 0.61–0.93, *P* = 0.008).

**Table 3:** Factors independently associated with overall survival in inoperable biliary tract cancers in SEER‐Medicare after multivariate adjustment

	Overall survival in chemotherapy cohort (*n* = 1053)	*P*	Overall survival in no chemotherapy cohort (*n* = 1290)	*P*
	Adjusted HR (95% CI)		Adjusted HR (95% CI)	
Receipt of radiotherapy	0.82 (0.70–0.97)	0.02	1.09 (0.91–1.30)	0.34
Age at diagnosis				
≤70	Reference	–	Reference	–
71–75	1.25 (1.06–1.47)	0.008	1.11 (0.92–1.35)	0.29
76–80	1.25 (1.05–1.49)	0.01	1.24 (1.03–1.51)	0.03
>80	1.48 (1.22–1.82)	<0.0001	1.25 (1.05–1.49)	0.01
Tumor stage (SEER historic stage)				
Local	Reference	–	Reference	–
Regional	1.03 (0.85–1.25)	0.77	1.21 (1.05–1.39)	0.009
Metastatic	1.47 (1.21–1.78)	0.0001	1.84 (1.58–2.14)	<0.0001

HR, hazard ratio; CI, confidence interval; SEER, Surveillance, Epidemiology, and End Results.

We found similar results when we analyzed cancer‐specific survival. In patients who received chemotherapy, receipt of radiation was associated with improved cancer‐specific survival, with an adjusted hazard ratio of 0.82 (95% 0.69–0.98, *P* = 0.03). In patients who did not receive chemotherapy, receipt of radiation was not associated with improved cancer‐specific survival, with an adjusted hazard ratio of 1.08 (95% 0.90–1.31, *P* = 0.40).

We also evaluated whether our results differed among patients in the chemotherapy cohort who received specifically gemcitabine and cisplatin, and found that after adjusting for this regimen, radiation was still associated with an overall and cause‐specific survival benefit, with adjusted hazard ratios of 0.81 (95% CI 0.68–0.95, *P* = 0.01) and 0.80 (95% CI 0.67–0.95, *P* = 0.01), respectively. The gemcitabine and cisplatin regimen was associated with improved overall and cause‐specific survival, with adjusted hazard ratios of 0.82 (95% CI 0.68–0.99, *P* = 0.04) and 0.76 (95% CI 0.62–0.93, *P* = 0.008), respectively. Finally, we found that IMRT/SBRT was not associated with improved survival among those who received radiotherapy.

We performed a propensity‐scored matched analysis in those receiving chemotherapy and those who did not, and found similar results (Appendix C).

### Differences between subjects with and without chemotherapy

Patients who did and did not receive chemotherapy differed with respect to receipt of radiation therapy as well as personal characteristics. Compared to those who did not, patients receiving chemotherapy were more likely to be treated with radiation and to be young, married, White, diagnosed with regional/metastatic tumors, diagnosed in later calendar years, residing in metropolitan areas without state buy‐in, have good functional status and have a biliary stent or drain (Table 4).

**Table 4:** Factors independently associated with receipt of chemotherapy in multivariate logistic model

Characteristic	OR for Receipt of Chemotherapy	95% CI	*P*
Age at diagnosis			
≤70	Reference	–
71–75	0.73	0.57–0.95	0.02
76–80	0.57	0.44–0.75	<0.0001
>80	0.23	0.18–0.31	<0.0001
Race			
White	Reference	–
Black	0.66	0.47–0.94	0.02
Other^a^	0.74	0.54–1.03	0.07
Marital status			
Married	Reference	–
Single^b^	0.74	0.61–0.90	0.002
Disability status			
Good	Reference	–
Poor	0.48	0.33–0.70	0.0001
SEER historic stage			
Localized	Reference	–
Regional	1.98	1.55–2.53	<0.0001
Metastatic	3.75	2.94–4.78	<0.0001
Year of diagnosis			
1998–2002	Reference	–
2003–2007	1.49	1.17–1.91	0.001
2008–2011	2.02	1.58–2.59	<0.0001
Rural/urban			
Metropolitan	Reference	–
Urban/rural	0.70	0.54–0.92	0.01
State buy‐in			
Yes	Reference	–
No	0.73	0.56–0.94	0.01
Radiation			
Yes	Reference	–
No	0.38	0.30–0.49	<0.0001
Stent/drain			
Yes	Reference	–
No	0.56	0.46–0.69	<0.0001

OR, odds ratio; CI, confidence interval; SEER, Surveillance, Epidemiology, and End Results.

a Other includes American Indian/Alaskan Native, Asian/Pacific Islander.

b Single includes unmarried, divorced, separated, and widowed.

## Discussion

Patients who present with inoperable biliary tract cancers have unfavorable prognosis and need effective therapies. However, the rarity of biliary tract tumors makes it challenging to study optimal treatment approaches through adequately powered trials. The large sample size and real‐world settings make large, administrative databases appealing for filling the knowledge gap of optimal management in diseases with poor prognosis and few effective treatment options. Using SEER‐Medicare, we found that radiotherapy at diagnosis in elderly patients with inoperable biliary cancers is associated with a survival benefit among those with nonmetastatic disease who receive chemotherapy.

Although chemotherapy is the standard of care in patients with inoperable biliary tract cancers, the role of radiotherapy is less established. The ABC‐02 trial found that gemcitabine plus cisplatin was associated with superior overall survival (11.7 months vs. 8.1 months) compared to gemcitabine alone in treatment‐naive patients with advanced biliary cancers 5. While there has been no randomized evidence for radiotherapy, a phase 2 study showed that radiation with concurrent hepatic artery floxuridine was associated with improved survival over historical controls 12. The largest series suggesting a survival benefit with radiotherapy in inoperable biliary tract tumors include several SEER analyses of intrahepatic and extrahepatic cholangiocarcinomas 14, 15, 42. However, these studies were unable to control for chemotherapy and performance status, as these data are not available in SEER, and they did not address immortal time bias 25, 26, 27, 28, 29 as a possible explanation of the finding of more favorable survival with radiotherapy. The existence of this bias has been well‐reported in the literature: patients who receive treatment must have survived long enough to receive treatment, thus resulting in survival estimates that are biased in favor of treatment.

We were able to use Medicare claims to determine receipt of chemotherapy and to approximate comorbidity and performance status. Additionally, the timing of radiotherapy administration was ascertained through claims, allowing us to address immortal‐time bias using a landmark analysis 25, 27, 43 that excludes patients who die too soon to receive radiotherapy. After addressing immortal time bias and controlling for chemotherapy and performance status, we did not find a statistically significant survival benefit for the entire cohort, as has been observed in prior studies.

We were able to assess the differential effect of radiotherapy in those who received chemotherapy and those who did not, and found that any survival benefit of radiation appeared confined to those who received chemotherapy. Given that intrahepatic cholangiocarcinomas, extrahepatic cholangiocarcinomas, and gallbladder cancers differ in terms of epidemiology, molecular pathogenesis and patterns of spread 44, 45, we evaluated whether radiation's efficacy differs by tumor type, and did not find a difference. Finally, we found that patients with nonmetastatic disease were the ones most likely to derive a survival benefit, whereas those with metastatic disease did not benefit.

One possible explanation for the present findings is that the improved control of systemic disease with chemotherapy allows the local effects of radiotherapy to translate into longer survival. Alternatively, patients who received chemotherapy were younger, healthier, and with better overall prognosis than those who did not, so for them, radiotherapy may have been selected with definitive rather than palliative intent. Given that Tao et al. recently demonstrated that radiation dose impacts survival in patients with unresectable intrahepatic cholangiocarcinoma 16, using higher doses of radiation could have contributed to survival benefit. Our finding that the addition of radiotherapy was not associated with a survival benefit among those with metastatic disease supports this explanation, as these patients were more likely treated with palliative courses of radiotherapy. Although palliative radiotherapy may improve stent patency and symptoms 10, 46, 47, it may not necessarily lead an observable survival benefit in these patients. Unfortunately, a limitation of administrative claims datasets is the lack of important treatment details, such as treatment intent and radiation dose, so we can only speculate on reasons behind this survival benefit.

Although we controlled for available patient, tumor, treatment, and socioeconomic characteristics in multivariate and propensity score analyses, and found consistent results with both approaches, we were unable to account for unmeasured confounders. More granular tumor characteristics such as histologic grade, tumor size and multifocal disease were not reliably available for all patients due to the lack of definitive, surgical staging. We chose to use the SEER historic staging, which was most consistently recorded over time and across tumor types. Thus, it is possible that patients with more aggressive tumors were more likely to receive radiotherapy, and that these adverse disease characteristics were not entirely captured in our study.

Additional limitations of this study include the possibility that performing a landmark analysis can lead to loss of power as well as not allow evaluation of the early impact of therapy by omitting early events. Although the potential to miss early treatment benefit with a landmark analysis is greater in aggressive diseases such as biliary tract cancers because of the high number of early events, the magnitude of immortal time bias is also greater because overall survival is short. Finally, although we found that early radiation, given within 4 months of diagnosis, is associated with a survival benefit compared to those who received no or late radiation, we did not specifically evaluate the treatment approach of delayed radiation. Given the risk of systemic spread, a common treatment approach in advanced biliary tract cancers is to start with chemotherapy in order to address micrometastases as well as to determine the natural history of the disease, and to reserve radiation for patients who do not progress after a sufficient number of chemotherapy cycles 13.

Despite the survival benefit associated with radiotherapy among those who received chemotherapy, we found that the use of radiation has declined, while the use of chemotherapy increased during that same period. Specifically, we found that patients who were poor, single, or elderly were less likely to receive radiotherapy. We found that many of these same demographic and socioeconomic factors were also independently associated with decreased receipt of chemotherapy, which is more accepted as standard of care treatment. Disparities in cancer treatment and outcomes based on age, race, socioeconomic and marital status have been commonly reported 48, 49, 50, 51. With improvements in systemic therapy, it is likely that radiotherapy will play a larger role in the treatment of unresectable disease, particularly as advancements in radiotherapy techniques allow for increased dose intensity and reduced normal tissue toxicity 52.

## Conclusion

The addition of radiotherapy to chemotherapy is associated with improved survival in elderly patients with unresectable biliary tract tumors, primarily in nonmetastatic patients, and supports reserving radiotherapy for select patients who receive chemotherapy. With improvements in systemic therapy and radiation technique including the use of intensity‐modulated and stereotactic body radiotherapy, radiotherapy may play an increasingly important role in impacting survival. Further study is needed to determine how best to optimize therapy in these patients and to improve access to this therapy.

## Conflict of Interest

None declared.

## Figures and Tables

**Figure C1:**
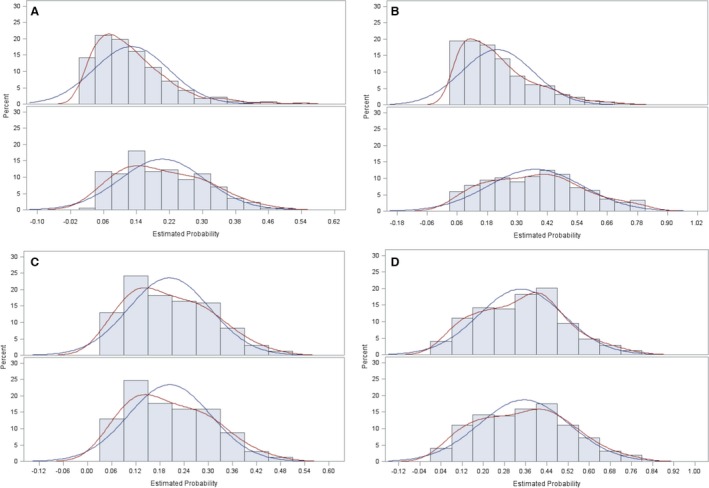
Distribution of propensity scores of radiation (top) and no radiation groups (bottom) (a) before matching in no chemotherapy group, (b) before matching in chemotherapy group, (c) after matching in no chemotherapy group and (d) after matching in chemotherapy group.

## Appendix A

The SEER‐Medicare database links information from the SEER tumor registries and the Centers for Medicare and Medicaid Services (CMS) Medicare claims data. SEER includes individual cancer registries across the United States, covering approximately 28% of the US population, and includes information about tumor stage, patient demographics, and first course of treatment. These registry data are linked to Medicare claims files by encrypted patient identifiers. The Medicare program provides payment records for hospital, physician and outpatient medical services for 97% of US citizens who are 65 years or older.

We identified patients with invasive biliary tract tumors diagnosed between 1998 and 2011 using topographic and morphology codes 53 (Table A1). We excluded patients who had undergone resection, identified using SEER coding for surgery of the primary site. We also excluded patients who had prior malignancies or unknown stage. In order the ensure the completeness of Medicare claims records, we restricted our cohort to patients who had continuous enrollment in Medicare Part A and Part B during the 16 month period from 12 months prior to diagnosis to 4 months after diagnosis. This restriction allowed ascertainment of comorbidity status as well as initial treatment at diagnosis. In addition, the restriction excluded patients who died within 4 months of diagnosis to address potential immortal time bias 25, 26, 27, 28, 29. Patients who died within this time would not have had the opportunity to undergo and benefit from radiation. This interval was chosen a priori to be broad enough after diagnosis to allow receipt of radiotherapy, but narrow enough so as to avoid counting radiation courses for disease progression or recurrence. We also excluded patients who were enrolled in a non‐Medicare health maintenance organization during this time period because their billing claims are not submitted to Medicare for reimbursement. Similarly, we excluded patients without any claims during the month of diagnosis as they likely had other insurance that covered their cancer work‐up and treatment. Table A2 summarizes our cohort selection process.

**Table A1:** Topographic and morphology codes for biliary tract tumors used in cohort selection

Tumor type	Topographic code (anatomic site)	Morphology
Extrahepatic cholangiocarcinoma	C24.0	8000, 8001, 8010, 8020, 8021, 8022, 8140, 8160, 8161, 8260, 8261, 8263, 8480, 8481, 8490, 8500, 8560
	C24.1	8160
	any	8162, 8163
Intrahepatic cholangiocarcinoma	C22.0	8160, 8161
	C22.1	8000, 8001, 8010, 8140, 8160, 8161, 8310, 8480, 8481, 8500, 8490
Galbladder	C23.9	8000, 8001, 8010, 8020, 8021, 8022, 8140, 8260, 8261, 8263, 8480, 8481, 8490, 8560, 8570

**Table A2:** Cohort selection algorithm for SEER‐Medicare analyses

Steps	No.	Percent
Biliary tract tumor histology	18,984	100%
Surgery status known and did not undergo surgery for biliary tract cancer	11,458	60%
Invasive cancer, no prior malignancy and known stage	8022	42%
Had Medicare Part A/B coverage and were not enrolled in any HMO during the 16‐month period starting 12 months before and 4 months after diagnosis[Link]	2406	13%
No claims found during diagnosis period	2343	12%

This excludes patients who died within 4 months of diagnosis (for landmark analysis) because these patients would not have had coverage.

## Appendix B Claim codes.

**Table:**

Type	HCPCS/CPT	ICD‐9	Revenue center	National drug code (DME files)
Chemotherapy administration	J8500‐J9999, J7150, 964xx, 965xx, Q0083‐Q0085, C8953‐5, C9414‐37, G0355‐62, S0115‐6, S9329‐31	V58.1, V66.2, V67.2, 99.25	0331, 0332, 0335	00004110020, 00004110150, 00004110116, 00004110051, 00004110013, 00004110022, 00004110113, 00004110151, 00004110175
Radiotherapy delivery codes	C1715‐C1720, C2616, C2632‐C2640, C2698‐9, 0073T, 0082T, G0173‐4, G0243, G0251, G0339‐40, 77225, 77371‐3, 77418, 77401‐77431, 77469‐70, 77499, 77520, 77522‐3, 77750, 77761‐77763, 77776‐77778, 77781‐77787, 77789‐90, 77799	V58.0, 92.2, 92.3, 92.4	333	–
Biliary stent/drain	43260‐9, 43271‐2, 74320, 74328‐30, 74363, 75980, 75982, 47510‐1, 47525, 47530, 47555, 47556	51.10‐1, 51.64, 51.84‐7, 51.9, 51.98‐9, 52.13, 52.93, 87.51	–	–
Influenza shot	90658, G0008	V0481	–	–

## Appendix C Propensity‐score analyses.

As we found a significant interaction between radiation and chemotherapy in our Cox multivariate model, we analyze those who received chemotherapy and those who did not receive chemotherapy in separate propensity‐score matched analyses. Propensity scores were estimated using a logistic regression model with radiation treatment as the dependent variable and covariates that would affect both treatment selection and outcome as independent variables (Table 1). Patients were then matched 1:1 without replacement, using a greedy matching algorithm 54 with a maximum caliper of 0.2 times the standard deviation of the logit of the propensity score 55. By matching on the propensity score, we hoped to identify radiated and non‐radiated patients with a comparable distribution of observed covariates, We restricted the analysis to observations with a propensity score range that was common to both treated and untreated patients, excluding all patients in the non‐overlapping parts of the propensity score distribution.

Of the 274 patients who received both radiation and chemotherapy within the first 4 months of diagnosis, 254 patients were matched to a control who received chemotherapy but no radiation. We were able to match 170 patients of the 177 patients who received only radiation and no chemotherapy within the first 4 months of diagnosis to a patient who did not receive radiation or chemotherapy. We then examined balance in baseline covariates using standardized differences. Because many of our baseline variables were categorical, we used a multivariate Mahalanobis distance method to generalize the standardized difference metric to handle a multinomial sample 56. Standardized mean differences of all the covariates were overall reduced after matching (Table C1). The distributions of the propensity scores before and after matching are shown in Figure C1 for chemotherapy and no chemotherapy cohorts respectively.

**Table C1:** Absolute standardized differences (averaged over imputed datasets) of all covariates before and after matching

Characteristic	Chemotherapy		No chemotherapy	
	Before matching	After matching	Before matching	After matching
	Absolute standardized differences			
Age at diagnosis	0.17	0.09	0.30	0.03
Gender	0.21	0.008	0.09	0.07
Race	0.16	0.03	0.15	0.00
Marital status	0.19	0.02	0.24	0.08
Comorbidity index	0.03	0.02	0.03	0.10
Disability status	0.04	0.03	0.28	0.09
Tumor type	0.47	0.09	0.29	0.07
SEER historic stage	0.61	0.10	0.24	0.06
Year of diagnosis	0.40	0.09	0.08	0.06
SEER registry	0.17	0.08	0.10	0.11
SES composite index	0.13	0.05	0.10	0.08
Rural/urban	0.04	0.00	0.07	0.07
State buy‐in	0.12	0.09	0.30	0.08
Stent/drain	0.63	0.04	0.19	0.03

Among those who received chemotherapy, radiotherapy was associated with improved overall survival, with a hazard ratio of 0.77 (95% CI 0.65–0.92, *P* = 0.004). Among those who did not receive chemotherapy, radiotherapy was not associated with improved overall survival, with a hazard ratio of 0.97 (95% CI 0.79–1.21, *P* = 0.81).
